# 

**Published:** 1915-05

**Authors:** 


### Deaths.

Dr. Harney Upshaw died April 2, 1915, at his home in Somerville.

Dr. J. T. Bolton, pioneer planter, physician and Confederate veteran, died March 29, 1915, at his home in Wharton, Texas.

Dr. Pipkin, familiarly known as "Grandfather Pipkin," died

March 25, 1915, at the home of his son, Dr. G. P. Pipkin, of Lancaster, Texas.

Dr. E. M. Legg died March 6, 1915, at his home in Abernathy, Texas.

Dr. J. M. Corbin died March 31, 1915, at the home of his father in Sherman.

Dr. W. F. Killian, for many years a resident of Buda, died at his home April 9, 1915.

Dr. W. D. Patton died April 5, 1915, at his home in Amarillo.

Dr. G. C. Abell, owner of Abell's Sanitarium, Texarkana, died very suddenly at his home in that city April 10th.

Dr. P. S. Smith, formerly of Comanche, died at the home of his mother in Aplin, Ark., March 29, 1915.

Dr. I. H. Herring died at his home in Mexia January 13, 1915.

Dr. McElroy, of Reily Springs, died at the home of his son-in-law, J. B. Melton, at Union, April 11, 1915.

---

The farmer, wearing a long face, entered the country drug store. "I've got something wrong with my stomach," he announced, "and I want you to give me something for it."

"All right," replied the apothecary cheerfully; "what are your symptoms?"

"Every little while something seems to rise up and settle back, and then by and by it rises up and settles back again."

The druggist stroked his chin reflectively. "Look here," he said gravely; "you haven't gone and swallowed an elevator, have you?"—*Dallas News.*

